# Water content of the endothelial glycocalyx layer estimated by volume kinetic analysis

**DOI:** 10.1186/s40635-020-00317-z

**Published:** 2020-07-10

**Authors:** Robert G. Hahn

**Affiliations:** 1 Research Unit, Södertälje Hospital, Södertälje, Sweden; 2 Karolinska Institutet at Danderyds Hospital (KIDS), Stockholm, Sweden

**Keywords:** Glycocalyx, Physiology, Pharmacokinetics, Plasma volume, Analysis, Ringer’s solution

## Abstract

**Background:**

The water volume of the endothelial glycocalyx layer has been estimated at 0.7 to 1.7 L using tracer methods of unclear value. The present study attempts to measure this fluid volume by analyzing the kinetics of a crystalloid fluid load.

**Methods:**

An intravenous infusion of approximately 1 L of Ringer’s acetate was administered to 35 healthy volunteers, and the central volume of distribution of the water volume was calculated from the urinary excretion and frequent measurements of the fluid-induced hemodilution using mixed-effects modeling software. Comparisons were made with the plasma volume derived from three published anthropometric regression equations based on isotope measurements. In a second analysis, up to 2.5 L of Ringer’s was administered to 60 volunteers selected from a cohort of 160 to have as similar hematocrits as possible to the volunteers whose data were used to create the anthropometric equations.

**Results:**

Volume kinetics showed that the infused crystalloid fluid occupied a larger central fluid space than was estimated with the isotope measurements. The first analysis of the 35 subjects indicated a mean difference of 0.51 L in males and 0.49 L in females. The second, larger analysis showed a mean excess volume of 0.43 L, which was approximately 15% of the circulating plasma volume.

**Conclusions:**

A crystalloid fluid load expands a 0.4–0.5 L larger central fluid space than the circulating plasma volume. The excess volume is probably located in the glycocalyx layer.

## Background

The endothelial glycocalyx is a layer of heavily glycosylated proteins that covers the luminal side of the endothelium throughout the cardiovascular system and the lymphatics. The glycocalyx has a thickness of 0.2–5 μm and plays a role in local vasodilatation, coagulation, and inflammation [1, 2]. This layer is sometimes referred to as a “vascular barrier,” as it prevents excessive capillary leakage of macromolecules [3].

Degradation of the glycocalyx layer “shedding” occurs in inflammatory states, during ischemia, and after vigorous volume loading, and shedding changes the physiology of the endothelium [2, 3]. The glycocalyx also contains water that can probably be released to the flowing plasma in response to hemorrhage or acute elevations of the plasma oncotic pressure [4] although degradation of the glycocalyx also increases the capillary permeability for macromolecules, which promotes hypovolemia.

The volume of the water reservoir residing inside the glycocalyx meshwork has been the subject of debate, but it has been estimated at 700 mL by indocyanine green measurements [5] and at 1.7 L by low molecular dextran measurements [6]. Research suggests that the glycocalyx has a capacity to rapidly increase the plasma volume (PV) by as much as 50%, but the methodologies used to confirm this have been questioned [7, 8]. More data are needed to clarify how much water the glycocalyx contains.

**Fig. 1:**
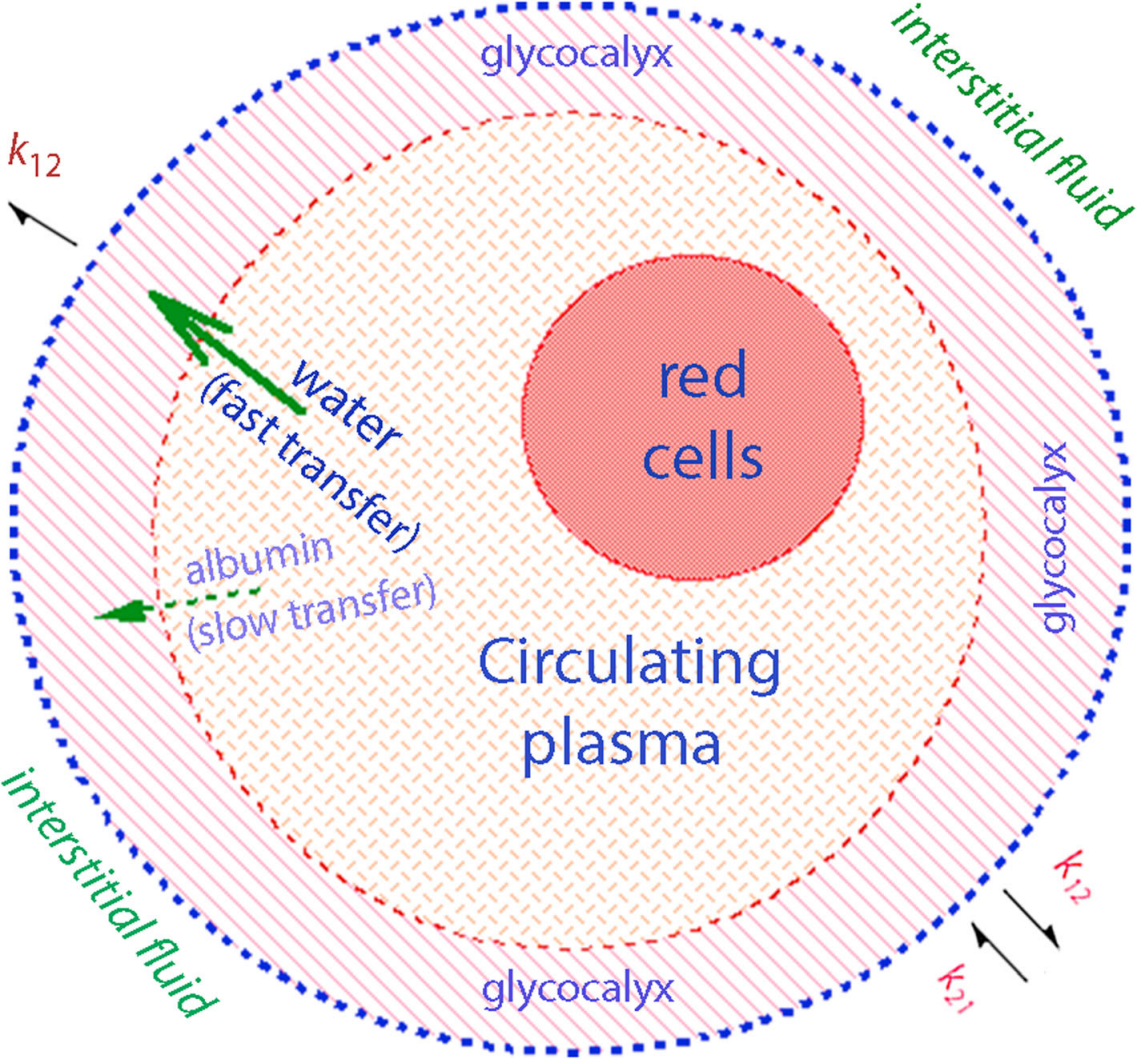
Schematic drawing of circulation in which red blood cells and plasma is closed off from the glycocalyx layer while infused fluid penetrates easily. Albumin enters the glycocalyx slowly and finally leaks out to the interstitial fluid via endothelial clefts. The capillary leakage of fluid occurs much faster and, at any moment, is the product of the volume expansion of the plasma and the glycocalyx layer and the rate constant *k*_*1*2_. The so-called F-cell ratio might be due to the difference between red blood cells and plasma with regard to their access to the glycocalyx layer

The aim of the present study was to quantify the water volume hidden within the glycocalyx by comparing the tracer techniques used to estimate the blood volume [9] versus a population kinetic analysis of crystalloid fluid [10]. The rationale is that red blood cells are excluded from the glycocalyx, whereas an infused fluid volume will also penetrate into this layer (Fig. 1). Three anthropometric equations for the blood volume are then compared to the penetration of crystalloid fluid into the glycocalyx, as determined by population volume kinetic analyses in healthy adult males and females given an intravenous (i.v.) infusion of fluid.

## Methods

Infusion experiments were selected from a database of 160, in which Ringer’s acetate had been administered i.v. to fully conscious euhydrated healthy adult volunteers. The database was created based on a 20-year effort to study the volume kinetics of infusion fluids by measuring the blood hemoglobin (Hb) concentration in a standardized way at precisely timed intervals (5–15 min) during and after fluid administration.

In a first round, 35 experiments were selected to avoid marked cardiovascular and hormonal responses to vigorous PV expansion. Therefore, criteria for inclusion were set as infusion rates < 50 mL/min, infused volume < 1500 mL, and a body weight of 50–90 kg.

In a second round, 60 experiments were selected from the 160 to include volunteers with hematocrit being as close as possible to the studies from which the anthropometric equations were taken. The fluid volume was not restricted, and many volunteers received > 1500 mL of Ringer’s acetate (range 724–2488 mL).

All studies had been approved by the appropriate local Ethics committee and were started between 8 and 9 am. Samples were collected from a cannula placed in one arm vein, while Ringer’s was infused into the other arm. The urinary excretion was measured when the volunteers voided spontaneously, as well as at the end of each experiment.

### Kinetic analysis

Volume kinetics is an adaptation of pharmacokinetic modeling for infusion fluids but is based on hemodilution rather than on drug concentrations [10].

**Fig. 2:**
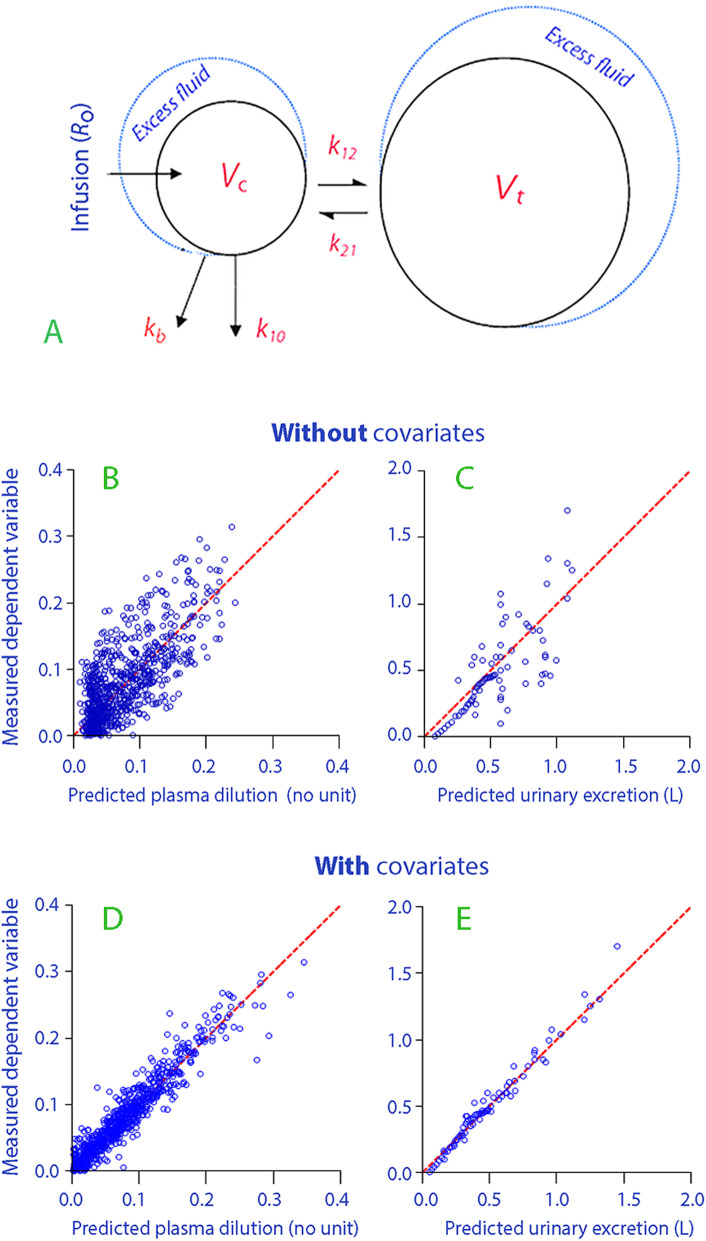
Schematic drawing of the kinetic model (a). Measured versus model-predicted plasma dilution and urinary excretion without (**b**, **c**) and with (**d**, **e**) consideration of the covariates of infusion rate and body weight

A two-volume kinetic model created to reflect body physiology was used (Fig. 2a). Here, the fluid is infused at a rate *R*_o_ into an expandable body fluid space *V*_c_ (the plasma) from which distribution and elimination is governed by the three rate constants: *k*_12_ for flow from *V*_c_ to a peripheral space, *V*_t_ (interstitial space), *k*_21_ for flow in the opposite direction (from interstitium to plasma), and *k*_10_ for elimination by urinary excretion. All flows are proportional, by a rate constant, to the volume expansion of a fluid space. This fluid space is *V*_c_ for *k*_12_ and *k*_10_, and *V*_t_ for *k*_21_. *V*_c_ is a scaling factor between plasma dilution and PV expansion, but it can also be used to represent the PV before the infusion takes place [10].

This two-volume model was fitted to the two dependent variables, which were the urinary excretion and the frequently measured plasma dilution. The changes in Hb were converted to plasma dilutions by the expression [(Hb_o_/Hb) − 1)/(1 − hematocrit_o_)], where the subscript “o” denotes a baseline measurement. The rate constant *k*_10_ was taken as the urinary excretion divided by the volume expansion of *V*_c_ over time.

In summary, the equations used in the calculations were:

$$ {\displaystyle \begin{array}{l}\mathrm{d}{v}_{\mathrm{c}}/\mathrm{dt}={R}_{\mathrm{o}}-{k}_{12}\left({v}_{\mathrm{c}}-{V}_{\mathrm{c}}\right)+{k}_{21}\left({v}_{\mathrm{t}}-{V}_{\mathrm{t}}\right)-{k}_{10}\left({v}_{\mathrm{c}}-{V}_{\mathrm{c}}\right)\\ {}\mathrm{d}{v}_{\mathrm{t}}/\mathrm{dt}={k}_{12}\left({v}_{\mathrm{c}}-{V}_{\mathrm{c}}\right)-{k}_{21}\left({v}_{\mathrm{t}}-{V}_{\mathrm{t}}\right)\\ {}\left({v}_{\mathrm{c}}-{V}_{\mathrm{c}}\right)/{V}_{\mathrm{c}}=\left[\left({\mathrm{Hb}}_{\mathrm{o}}/\mathrm{Hb}\right)-1\right)\Big]/\left(1-{\mathrm{hematocrit}}_{\mathrm{o}}\right)\\ {}{k}_{10}=\mathrm{urinary}\ \mathrm{excretion}/\mathrm{area}\ \mathrm{under}\ \mathrm{the}\ \mathrm{curve}\ \mathrm{for}\ \left({v}_{\mathrm{c}}-{V}_{\mathrm{c}}\right)\end{array}} $$

All data were entered into the Phoenix software for nonlinear mixed effects, version 1.3 (NLME, Pharsight, St. Louis, MO) and analyzed on a single occasion. The search routine used was the First-Order Conditional Estimation Extended Least Squares (FOCE ELS), which is slow but yields precise estimates of the unknown parameters (*V*_c_, *k*_*1*2_, *k*_21_, and *k*_10_) in the model. If desired, the size of *V*_t_ can be estimated as *V*_c_*k*_12_/*k*_21_.

### Covariates

Covariates were added in sequence, as guided by a reduction of the residuals for the model when re-creating the input. The considered covariates were gender, body weight, height, cube of height, infusion rate, infusion volume, and infusion time. A covariate was accepted if the 95% confidence interval (CI) of its estimate did not include zero and the residual for the model, expressed as − 2(LL) (log likelihood), was reduced by *P* < 0.05. The parameters ultimately reported represented a “Full block model,” in which the inter-individual variability of the fixed effects and their covariates (theta:s) are supplemented by the variances and the covariance variability of the random effects (eta:s) [11, 12]. The full block model generates the most precise simulations.

### Blood volume

The blood volume (BV) of each volunteer was calculated by inserting their gender, height (H), and body weight (BW) into regression equations derived based on measurements performed in populations of volunteers.

Allen et al. [13] obtained the BV in 81 subjects from the hematocrit and measurement of the PV using a T-1824 dye technique:

$$ {\displaystyle \begin{array}{l}\mathrm{BV}=0.417\ {\mathrm{H}}^3+0.0450\ \mathrm{BW}-0.03\ \left(\mathrm{males}\right)\\ {}\mathrm{BV}=0.414\ {\mathrm{H}}^3+0.0328\ \mathrm{BW}-0.03\ \left(\mathrm{females}\right)\end{array}} $$

Nadler et al. [14] calculated BV in 155 men and women with widely ranging body weights and ages using albumin molecules tagged with radioactive iodine:

$$ {\displaystyle \begin{array}{l}\mathrm{BV}=0.3669\ {\mathrm{H}}^3+0.03219\ \mathrm{BW}+0.6041\ \left(\mathrm{males}\right)\\ {}\mathrm{BV}=0.3561\ {\mathrm{H}}^3+0.03308\ \mathrm{BW}+0.1833\ \Big(\mathrm{females}\end{array}} $$

Retzlaff et al. [15] recruited 78 volunteers and measured their red cell volumes with chromium-tagged erythrocytes and the PV with albumin marked with Evan’s blue dye:

$$ {\displaystyle \begin{array}{l}\mathrm{BV}=31.9\ \mathrm{H}+26.3\ \mathrm{BW}-2402\ \left(\mathrm{males}\right)\\ {}\mathrm{BV}=56.9\ \mathrm{H}+14.1\ \mathrm{BW}-6460\ \left(\mathrm{females}\right)\end{array}} $$

Allen and Nadler expressed height in meters, while Retzlaff used centimeters. Allen and Nadler but not Retzlaff corrected their equations for a hematocrit factor of 0.91 [16].

### Plasma volume

Further calculations compared PVs. The rationale underlying the use of a regression equation for BV and subsequent conversion of the result into the PV is that the neurohumoral control mechanisms in the body strive to maintain a stable BV, whereas the PV is poorly controlled. One set of regression equations for PV exist [15], but they must be applied at the same hematocrit as in the population that was used to create the regression equation to reflect the correct BV, which is the strictly controlled variable. For example, this priority is apparent in hemorrhage, where the body restores the BV while the PV is much less important [17]. The PV, by variations in hematocrit, also differs between populations and countries depending on nutrition and height above sea level.

The fluid kinetic model uses the plasma as a key variable because the plasma, rather than the blood, equilibrates with the other body fluid compartments.

Consistent use of the hematocrit was considered essential to allow comparisons between the two methods. Therefore, the same individual hematocrit value was applied in each volunteer to convert BV into PV. For this purpose, the BV estimated with the regression equations was multiplied by (1 − hematocrit_o_), while the Hb dilution was divided by (1 − hematocrit_o_).

### Statistics

Data are reported as the mean and standard deviation (SD), with kinetic parameters reported as the best estimate and 95% CI. *P* < 0.05 was considered statistically significant.

## Results

### Limited cardiovascular responses

Table 1 shows the demographics and the PV for the studied 14 males and 21 females, as obtained using the regression equations in the three published isotope studies.

**Table 1:** Demographic data and the plasma volumes predicted from three published studies

	Males	Females
*N*	14	21
Body weight (kg)	76 (8)	59 (7)
Height (cm)	180 (5)	165 (4)
Hematocrit (%)	41.2 (2.0)	36.9 (3.3)
Infusion rate (mL/min)	33.0 (12.6)	33.6 (12.5)
Infusion volume (mL)	1,075 (206)	1,175 (320)
Infusion time (min)	36 (12)	38 (16)
Plasma volumes		
Allen et al. [13]	3.42 (0.29)	2.38 (0.23)
Nadler et al. [14]	3.05 (0.22)	2.36 (0.23)
Retzlaff et al. [15]^1^	3.05 (0.22)	2.14 (0.21)
Mean	3.18 (0.23)	2.29 (0.23)

^1^Corrected for a hematocrit factor of 0.87 in the males and 0.90 in the females, as reported in the article

The kinetic analysis was based on measurements performed at 862 points in time (a mean of 25 per experiment).

The estimates of the model parameters are shown in Table 2. The ability of the base model to recreate the dependent variables is illustrated in Fig. 2 b and c. A second elimination compartment was tested (*k*_b_ in Fig. 2a), but it did not turn out to be statistically justified.

**Table 2:** Population kinetic parameters in the final model for infusion experiments conducted in 35 volunteers

	Covariate	Best estimate	2.5% CI	97.5% CI	CV%	− 2(LL)
Fixed parameters						
*V*_c_ (L)	-	3.12	2.49	3.76	10.4	
*k*_12_ (10^−3^ min^−1^)	-	35.4	16.3	54.5	27.5	
*k*_21_ (10^−3^ min^−1^)	-	12.8	4.6	21.0	32.7	
*k*_10_ (10^−3^ min^−1^)	-	23.2	16.6	29.8	14.5	− 2721
Covariate effect						
− *V*_*c*_	Body weight (kg)	1.16	0.14	2.17	44.8	− 2728
− *V*_*c*_	Infusion rate (mL/min)	0.52	0.28	0.77	23.9	− 2747
Full block model	All the above					− 2766

The right column shows how the log likelihood of the model gradually improves (decreases) when a new covariate is added. A reduction by 3.6 points is statistically significant by *P* < 0.05

*CI* confidence interval, *CV* between-subject coefficient of variation, *LL* log likelihood for the model

Two covariates affected *V*_c_, and their inclusion in the model markedly reduced the residuals (Fig. 2 d and e). The strongest covariate was the infusion rate, while the other was the body weight. Female gender was close to providing a significant rise in *k*_10_, but it fell out of the final model. The equation for *V*_c_ for an individual volunteer had the following form:

*V*_c_ (L) = 3.12 [(infusion rate)/33.4) ^0.52] [(body weight)/65.6) ^1.16]

where 33.4 is the mean infusion rate, and 65.9 is the mean body weight for all volunteers in this so-called “power model.”

**Fig. 3:**
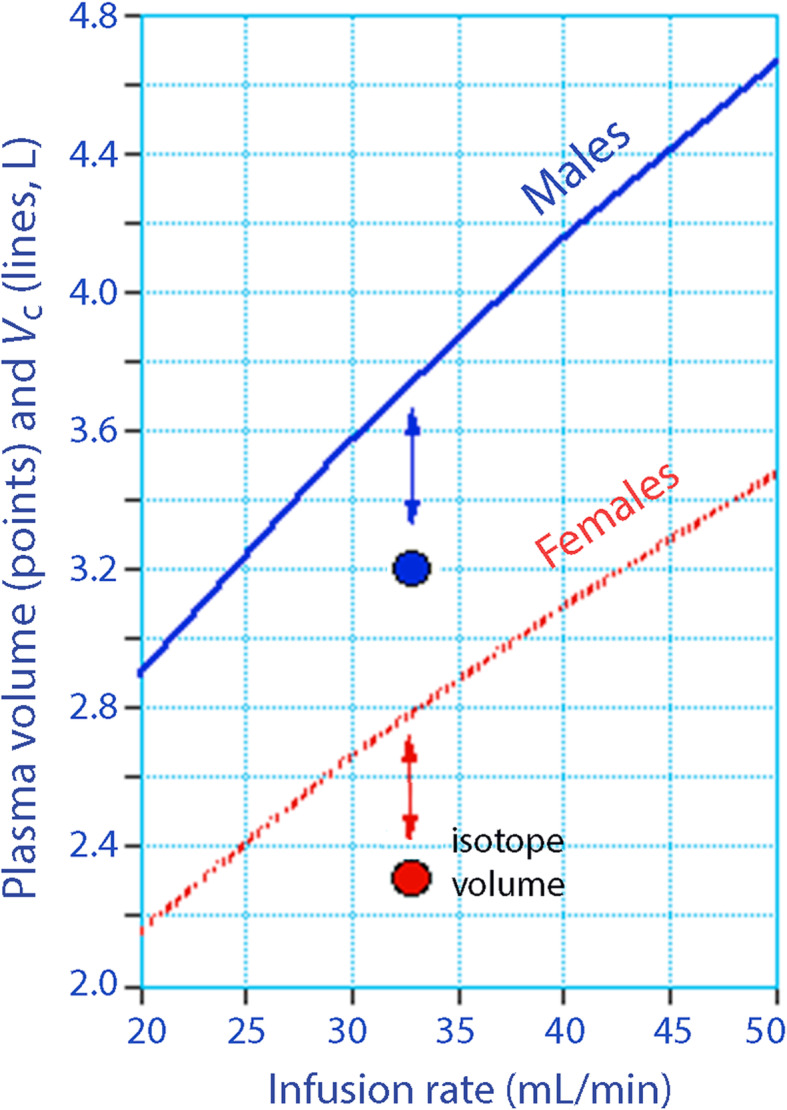
Central fluid space volume *V*_c_ for different infusion rates when the body weight is fixed to the mean for males and females (lines). The plasma volume is given as the mean of three published isotope studies using the gender, body weight, and height for each volunteer in the volume kinetic study (circles). The distance indicated by arrows is the water volume in the endothelial glycocalyx space

Two curves were constructed based on the equation for *V*_c_ shown above. At the mean infusion rates and body weights, the expected *V*_c_ would be 3.69 L for the males and 2.78 L for the females (Fig. 3).

The mean PV from the three published studies with tracers was 3.18 L and 2.29 L for males and females (Table 1).

This means that the volume of distribution for fluid outside the circulating plasma, which is taken as the glycocalyx volume, was 3.69 − 3.18 = 0.51 L in the males and 2.78 − 2.29 = 0.49 L in the females.

The mean value of the PV from the three regression equations in each volunteer was then subtracted from the post hoc individual estimates of *V*_c_, as given by the Phoenix program. This procedure showed that volume kinetics indicated a 494 mL (mean) larger central fluid space than was determined with the isotope studies.

### Aligned hematocrits

A second analysis includes a wider range of experiments where the hematocrits of the volunteers who underwent the fluid infusions were within 3% of the mean value in the works of Allen et al. [13] and Retzlaff et al. [15]. However, Nadler et al. [14] did not report the hematocrit. The mean hematocrit was 46.3% for males while the mean hematocrit for women was 40.5% (39.3% for Allen et al*.* and 41.6% for Retzlaff et al.). In general, the hematocrits in the infusion group were lower than in the published works.

The cohort consisted of 40 males and 20 females who received 1695 (455) mL of fluid at a rate of 58.0 mL/min (63.2 in the males and 47.6 in the females). The mean infusion time was 30 min but varied between 15 and 80 min.

The body weight was 81.2 (9.7) kg in the males and 62.8 (9.3) kg in the females, and the corresponding heights 182.2 (5.3) and 166.6 (5.5) cm, respectively.

Sixteen patients (27%) were included also in the first analysis.

The new kinetic analysis was based on data collected at 1457 points in time (mean 24 per experiment). The output is shown in Table 3. Here, a second elimination function (*k*_b_) was statistically significant.

**Table 3:** Population kinetic parameters in the final model for infusion experiments in the extended analysis of 91 volunteers

	Covariate	Best estimate	2.5% CI	97.5% CI	CV%	− 2(LL)
Fixed parameters						
*V*_c_ (L)	-	3.54	3.14	3.93	5.7	
*k*_12_ (10^*−*3^ min^*−*1^)	-	73.6	58.7	88.5	10.2	
*k*_21_ (10^*−*3^ min^*−*1^)	-	59.6	49.9	69.3	8.3	
*k*_10_ (10^*−*3^ min^*−*1^)	-	20.3	17.4	23.1	7.1	− 4298
*k*_b_ (10^*−*3^ min^*−*1^)	-	11.2	8.1	14.3	14.2	− 4399
Covariate effect						
*V*_*c*_	Gender	− 0.33	− 0.48	− 0.19	− 22.2	− 4414
*V*_*c*_	Infusion rate (mL/min)	0.43	0.24	0.62	22.9	− 4432

*Infusion rate* is a power model with a mean infusion rate of 58.0 mL/min. *Gender* is an exponential model where male = 0 and female = 1. The latter becomes (e ^− 0.33). The full block model did not converge to yield 95% CI

*CI* confidence interval, *CV* between-subject coefficient of variation, *LL* log likelihood

The group estimate for *V*_c_, as corrected for the covariates, yielded a size of *V*_c_ of 3.67 L in the males and 2.34 L in the females.

Insertion of their demographic data and individual hematocrits into the three regression equations (Allen, Nadler, and Retzlaff) yielded an expected mean PV of 3.10 L in the males and of 2.18 L in the females.

The fluid volume outside the erythrocyte pool (i.e., the glycocalyx volume) then amounted to 3.67 − 3.10 = 0.57 L in the males and 2.34 − 2.18 L = 0.16 L in the females (mean for all subjects, 0.43 L).

The mean value of the PV from the three regression equations in each volunteer was then subtracted from the post hoc individual estimates of *V*_c_, as given by the Phoenix program. This procedure showed that volume kinetics indicated a 411 mL (mean) larger central fluid space than was determined with the isotope studies.

## Discussion

The present study demonstrated that the central volume of distribution for an infused crystalloid fluid in 35 healthy volunteers was approximately 500 mL larger than the PV determined with isotope-derived regression equations. An extended analysis of 60 volunteers showed this difference to average 430 mL, which is a similar result. Both estimates are smaller than the previously suggested size of the glycocalyx fluid volume, which were 700 mL [5] and 1.7 L [6].

The current data were analyzed by several modifications of the kinetic model, but the result appeared quite robust. The identified volume likely represents the water in the endothelial glycocalyx space, provided that infused crystalloid fluid equilibrates relatively quickly with this water reservoir.

### Between-subject variability

The two methods compared here have a certain degree of variability, mostly arising from between-subject variability. The standard error for the volume kinetic estimates of *V*_c_ was 5% (large cohort) and 10% (small cohort). The isotope methods are claimed to be associated with an error of up to 5% due to imprecise volume measurements [14]. Therefore, the combined standard errors associated with the methods used here are clearly too large to allow quantification of the glycocalyx volume in an individual subject with an acceptable degree of certainty.

The issue is quite different if we compare mean values of two methods when applied to the same subjects in large groups of volunteers. In that case, only the accuracy of each method is a potential problem because the between-subject variability and measurement errors due to precision are likely to cancel out. In fact, the accuracy of any biochemical analysis is of importance if we compare mean or median values between groups, while both the accuracy and precision are of concern if we compare individuals.

### Accuracy

Possible problems with the accuracy of the measurements of the glycocalyx volume in the present study can only be due to systematic analytical errors and misspecification of the kinetic models. However, the used tracer methods have long been considered the gold standard for measurement of BV and PV, and the currently used volume kinetic model was viewed as optimal for analysis of the dilution kinetics of crystalloid electrolyte fluids in several studies [10–12]. Alternative models have been attempted even here, but were deemed inferior.

### Unequal distribution of tracer

One potential source of model misspecification could be an unequal distribution of albumin and erythrocytes in the circulating BV. However, the molecules used for analysis by the two methods (Hb and/or albumin) were either identical or had the same kinetic profile; a recent analysis of 128 infusions shows that the plasma dilution and the PV expansion indicated by Hb and albumin are practically identical, provided that Hb is first corrected for the individually measured baseline hematocrit [18]. This illustrates that a normal capillary leakage of albumin is tightly balanced by albumin returning via the lymph. Therefore, aberrant intravascular distribution of erythrocytes and albumin in the large blood vessels is likely to affect both methods in a similar way. Body physiology has to be markedly disturbed (for example, by abrupt changes in arterial pressure) to cause the dilution kinetics of these two intravascular molecules to deviate from each other [19, 20].

### The isotope technique

The control methods consisted of i.v. injections of isotope-tagged erythrocytes, an albumin isotope, or a dye, followed by measurement of the plasma concentration after 10 min (and sometimes several times afterward) to account for the loss of tracer activity over time by backward extrapolation to zero time. The PV is then derived by the dilution principle (i.e., the distribution volume equals the dose divided by the plasma concentration at time zero).

Only one of the studies measured the erythrocyte mass while the other two inferred the total blood volume from the measured peripheral hematocrit. When the erythrocyte mass and PV are both measured, the relationship between them does not fully agree with the measured peripheral hematocrit. This discrepancy has been recognized since the 1930s and is called the “F-cell ratio” or “hematocrit factor” [16, 21]. The F-cell ratio is of no relevance to dilution kinetics, which only depends on the distribution volume of the infused fluid volume and not on how large a volume the tracer is distributed in.

The F-cell ratio has been interpreted in many ways. The hematocrit is uniform in large blood vessels [22] but is higher in the spleen and lower in the kidneys, heart, and capillary beds [21]. The lower hematocrit in the capillary bed is due to the Fåhraeus Effect, which is due to that erythrocytes flow only in the middle of vessels with a diameter that is 0.4 mm or less. Others believe that plasma tracers may be excessively lost from the circulation during the mixing phases [18] or that the F-cell ratio is due to cause to differences in flow rates between plasma and erythrocytes [23]. All these explanations are speculations, although some indirect data are usually presented to support them. Whatever the reason, the F-cell ratio implies that the infused albumin is distributed in a 10% larger space than would be expected from the assumption that all plasma and erythrocytes are located in a single well-stirred circulating volume.

This problem is overcome from a practical point of view by routine multiplication of the PV measurements by 0.91 before being reported [13, 20]. This conversion had already been adopted by two authors of the three regression equations used here [13, 14] while the F-ratio had to be implemented to the results obtained by the third set of equations [15]. Therefore, the measurements of PV with tracers should originally have been somewhat larger than the regression equations indicate. This “missing albumin space” could be the glycocalyx or a part thereof, provided that the injected albumin equilibrates with the water in this layer. There is experimental evidence that slow equilibration of albumin between the plasma and the glycocalyx layer actually occurs [24].

### Concentration versus dilution kinetics

The same molecule can be used to measure two different volumes, depending on whether concentration kinetics or dilution kinetics is applied. The circulating BV is estimated by erythrocytes if they are tagged with an isotope and re-injected into the blood. By contrast, the sum of the circulating BV and the glycocalyx volume is measured if crystalloid fluid is infused and the dilution of the same erythrocytes is calculated.

Hemodilution can indicate distribution volumes much larger than the BV because hemodilution closely mirrors the blood water concentration. Any short-term change in blood Hb shows the distribution of the water volume that equilibrates quickly with the circulating plasma [10]. The fact that erythrocytes do not pass into the glycocalyx layer has no relevance to the ability of hemodilution to detect this volume. By contrast, this limitation exists for measurements of BV with isotope-tagged erythrocytes.

The theory for the distribution of different molecules might be confusing, but the present study is based on the following assumptions. Erythrocytes occupy the circulating BV, while dilution of the erythrocyte concentration by fluid indicates the circulating BV plus the glycocalyx. Injected albumin occupies the circulating plasma after correction with the F-cell ratio (0.91). If the F-cell ratio corrects for the entrance of albumin to the glycocalyx layer or not is unclear,  but quite possible (Fig. 1).

### Volume kinetics as a method

All fluid in *V*_c_ constitutes a single well-stirred volume, which means that equilibration between the circulating plasma and the glycocalyx water must be more or less instant. Distribution of infused crystalloid fluid to the interstitial fluid space is another issue, and this process requires up to 30 min to be completed [11, 12]. In the kinetic model, distribution from the plasma to the interstitial fluid space is captured by the rate constant *k*_12_ and the return flow by the rate constant *k*_21_.

A pharmacokinetic model is not usually interpreted in physiological terms due to metabolism, so the distribution volumes may be more theoretical than anatomical. These considerations do not readily apply to infusion fluids. In fact, much evidence supports the idea that volume kinetics does reflect fluid distribution between the body fluid compartments. The elimination is conveniently quantified by the urine flow rate, and the *k*_21_-generated flow has an almost identical response time and flow pattern to that obtained by direct measurement in the thoracic duct [10]. The size of *V*_c_ is routinely close to the known PV, and many studies show that changes in hematocrit corresponds well to isotope-measured changes in plasma and blood volumes [25–27]. Transit times, partition coefficients, osmotic shifts, metabolism, and excretion have hampered the methods previously used to estimate the glycocalyx fluid volume [8], but these factors hardly apply to volume kinetics. However, experiments in non-steady state settings, or where the physiology is manipulated in close proximity to the infusion, should be avoided. They may provide very uncertain estimates of *V*_c_.

### Selection of volunteers

A large cohort of unselected volunteer infusion experiments seemed unsuitable for the present volume kinetic analysis. Therefore, the database with 160 experiments used here comprised only infusions given during non-stressed laboratory conditions in healthy volunteers. Nevertheless, the infusion volumes and rates still differed, and the subjects had vastly different body weights. The most accurate result would probably be obtained if vigorous PV expansion was avoided and the number of potentially important covariates was kept to a minimum. Therefore, a number of criteria were set up to create a more uniform group of only 35 experiments. Despite these criteria, the explorative add-on analysis, using a larger cohort with greater variability but with similar hematocrits, did not yield results that differed substantially from the first analysis. In both cases, the rate of infusion was a factor that increased *V*_c_, which can be interpreted to imply that a certain fluid pressure was needed to fill the glycocalyx with fluid and/or that the body weight was not sufficient to compensate for changes in PV due to body size. Use of the cube of height, which is applied in two of the regression equations, did not improve the model more than the body weight did.

### Site of the fluid excess

This evaluation consistently shows that the central volume of distribution for infused crystalloid fluid is approximately 500 mL larger than the circulating PV obtained by widely used regression equations corrected for the F-cell ratio. However, the exact site of this excess fluid cannot be determined with certainty. In this respect, this study shares the problem with previous attempts to quantify the water volume of the glycocalyx [5, 6], but the most likely explanation is still, at present, that the glycocalyx is the site of the excess water.

The glycocalyx therefore seems to increase its water volume by approximately 15% of the central volume expansion induced by volume loading, while 85% remains in the circulating plasma. In the first series of experiments, the maximum glycocalyx expansion would transiently average 60 mL. However, an even more likely view is that the glycocalyx layer is continuously flushed with plasma-mixed infusion fluid, which then passes through pores in the endothelial layer to enter the interstitial fluid space at a rate determined by the rate constant *k*_12_. Albumin would travel via the same route, but more slowly [24].

## Limitations

The present study is intended to enhance our knowledge about the sizes of the fluid spaces in the body, rather than suggesting a method for measurement of the glycocalyx water volume in individuals. The clinical importance of the glycocalyx water is primarily that this volume is released to the circulating plasma when the glycocalyx layer becomes degraded. This degradation can occur within 10 min in response to an appropriate stimulus, such as major hemorrhage, and the volume constitutes 15% of the circulating plasma, which is not negligible. However, glycocalyx degradation increases the capillary permeability of macromolecules. The resulting increase of the capillary leakage of plasma constituents promotes hypovolemia, which is a likely scenario after the initial transfer of non-circulating plasma to the circulating pool.

The excess fluid volume found here agrees with the “F-cell ratio” reported with isotope tracer techniques, although it is currently unclear if they represent the same excess volume. If they do, the excess water volume should have approximately the same albumin concentration as the circulating plasma.

The infused fluid, Ringer’s acetate, is slightly hypotonic (osmolality 270 mosmol/kg) and would therefore be expected to pass into the body cells and cause edema. However, the urine excreted in response to the fluid load has an initial sodium content much lower than that of the infused fluid, which counteracts this withdrawal [28].

The strength of the present comparisons is that the errors introduced by biological variability must be low, since the body weights, ages, lengths, and hematocrits were the same with both methods.

## Conclusion

The central volume of distribution for infused Ringer’s acetate in a group of 35 and another of 60 healthy volunteers was approximately 500 mL larger than the plasma volume obtained using isotope-based regression equations in three published studies. This volume difference might represent the water content of the endothelial glycocalyx layer.

## Data Availability

All data are available as Supplemental Excel files.

## References

[1] Pries AR, Secomb TW, Gaehtgens P (2000). The endothelial surface layer. Pflügers Arch.

[2] Bertram A, Stahl K, Hegermann J, Haller H, Hahn RG (2016). The glycocalyx layer. Clinical fluid therapy in the perioperative setting.

[3] Alphonsus CS, Rodseth RN (2014). The endothelial glycocalyx: a review of the vascular barrier. Anaesthesia.

[4] Woodcock TE, Woodcock TM (2012). Revised Starling equation and the glycocalyx model of transvascular fluid exchange: an improved paradigm for prescribing intravenous fluid therapy. Br J Anaesth.

[5] Rehm M, Haller M, Orth V, Kreimeier U, Jacob M, Dressel H, Mayer S, Brechtelsbauer H, Finsterer U (2001). Changes in blood volume and hematocrit during acute perioperative volume loading with 5% albumin or 6% hetastarch solutions in patients before radical hysterectomy. Anesthesiology.

[6] Nieuwdorp M, van Haeften TW, Gouverneur MCLG, Mooij HL, van Lieshout MHP, Levi M, Meijers JCM, Holleman F, Hoekstra JBL, Vink H, Kastelein JJP, Stroes ESG (2006). Loss of endothelial glycocalyx during acute hyperglycemia coincides with endothelial dysfunction and coagulation activation in vivo. Diabetes.

[7] Hahn RG (2015). Must hypervolaemia be avoided? A critique of the evidence. Anaesthesiol Intensive Ther.

[8] Michel CC, Curry FE (2009). Glycocalyx volume measurements: a critical review of tracer dilution methods for its measurement. Microcirculation.

[9] Gómez Perales JL (2015). Blood volume analysis by radioisotopic dilution techniques: state of the art. Appl Radiat Isot.

[10] Hahn RG (2020). Understanding volume kinetics. Acta Anaesthesiol Scand.

[11] Hahn RG (2017). Arterial pressure and the elimination of crystalloid fluid: a population-based study. Anesth Analg.

[12] Hahn RG (2017). Influences of the red blood cell count on the distribution and elimination of crystalloid fluid. Medicina.

[13] Allen TH, Peng MT, Chen KP, Huang TF, Chang C, Fang HS (1956). Prediction of blood volume and adiposity in man from body weight and cube of height. Metabolism.

[14] Nadler SN, Hidalgo JU, Bloch T (1962). Prediction of blood volume in normal human adults. Surgery.

[15] Retzlaff JA, Newton Tauxe W, Kiely JM, Stroebel SF (1969). Erythrocyte volume, plasma volume, and lean body mass in adult men and women. Blood.

[16] Chaplin HH, Mollison PL, Vetter H (1953). The body/venous hematocrit ratio: its constancy over a wide hematocrit range. J Clin Invest.

[17] Hahn RG, Brauer L, Rodhe P, Svensén CH, Prough DS (2006). Isoflurane inhibits compensatory intravascular volume expansion after hemorrhage in sheep. Anesth Analg.

[18] Hahn RG, Vincent J-L (2020). Do intensivists need to care about the Revised Starling Principle?. Annual update in intensive care and emergency medicine.

[19] Ewaldsson CA, Hahn RG (2005). Kinetics and extravascular retention of acetated Ringer’s solution during isoflurane and propofol anesthesia for thyroid surgery. Anesthesiology.

[20] Nemme J, Krizhanovskii C, Ntikia SO, Vanags I, Hahn RG (2019). Hypervolaemia does not cause shedding of the endothelial glycocalyx layer during hysterectomy; a randomised clinical trial comparing sevoflurane and propofol anaesthesia. Acta Anaesthesiol Scand.

[21] Lawson HC (1962) The volume of blood–a critical examination of methods for its measurement. In: Handbook of physiology. Sect 2. Circulation Vol I. Am Physiol Soc, Washington.

[22] Swan H, Wendell Nelson A (1971). Blood volume I: critique: spun vs. isotope hematocrit; ^125^RIHSA vs. ^51^CrRBC. Ann Surg.

[23] Michel CC, Arkill KP, Curry FE (2016) The revised Starling principle and its relevance to perioperative fluid therapy. In: Farag E, Kurz A (eds) Perioperative fluid management. Springer, Cham, Switzerland pp. 31-74.

[24] Vink H, Duling BR (2000). Capillary endothelial surface layer selectively reduces plasma solute distribution volume. Am J Physiol Heart Circ Physiol.

[25] Chien S (1958). Quantitative evaluation of the circulatory adjustment of splenectomized dogs to hemorrhage. Am J Physiol.

[26] Lister J, McNeill IF, Marshall VC, Plzak LF, Dagher FJ, Moore FD (1963). Transcapillary refilling after hemorrhage in normal man: basal rates and volumes; effect or norepinephrine. Ann Surg.

[27] Hahn RG (1987). A haemoglobin dilution method (HDM) for estimation of blood volume variations during transurethral prostatic surgery. Acta Anaesthesiol Scand.

[28] Hahn RG, Drobin D (2003). Rapid water and slow sodium excretion of Ringer’s solution dehydrates cells. Anesth Analg.

